# *APC* gene is modulated by *hsa-miR-135b-5p* in both diffuse and intestinal gastric cancer subtypes

**DOI:** 10.1186/s12885-018-4980-7

**Published:** 2018-10-30

**Authors:** Leandro Magalhães, Luciana Gonçalves Quintana, Dielly Catrina Favacho Lopes, Amanda Ferreira Vidal, Adenilson Leão Pereira, Lara Carolina D’Araujo Pinto, João de Jesus Viana Pinheiro, André Salim Khayat, Luiz Ricardo Goulart, Rommel Burbano, Paulo Pimentel de Assumpção, Ândrea Ribeiro-dos-Santos

**Affiliations:** 1 Laboratório de Genética Humana e Médica, Instituto de Ciências Biológicas, Universidade Federal do Pará, Belém, Brazil; 2 Núcleo de Pesquisas em Oncologia, Hospital Universitário João de Barros Barreto, Universidade Federal do Pará, Belém, Brazil; 3 Laboratório de Neuropatologia Experimental, Hospital Universitário João de Barros Barreto, Universidade Federal do Pará, Belém, Brazil; 4 Laboratório de Cultivo Celular, Faculdade de Odontologia, Instituto de Ciências da Saúde, Universidade Federal do Pará, Belém, Brazil; 5 Laboratório de Nanobiotecnologia, Instituto de Genética e Bioquímica, Universidade Federal de Uberlândia, Uberlândia, Brazil; 6 Laboratório de Citogenética Humana, Instituto de Ciências Biológicas, Universidade Federal do Pará, Belém, Brazil

**Keywords:** Gastric cancer, *Hsa-miR-29c-5p*, *Hsa-miR-135b-5p*, 3D culture, DNMT3A, CDC42, APC

## Abstract

**Background:**

Several genetic and epigenetic alterations are related to the development and progression of Gastric Cancer (GC), one of those being the deregulated microRNA (miRNA) expression profile. miRNAs are small noncoding RNAs that negatively regulate the expression of thousands of genes, including oncogenes and tumor suppressor genes. Our group identified, in previous studies, some miRNAs that are differentially expressed in GC when compared to the gastric mucosa without cancer, including *hsa-miR-29c* and *hsa-miR-135b.* The aim of the study was to modulate the expression of the miRNAs *hsa-miR-29c-5p* and *hsa-miR-135b-5p* and evaluate the expression of their target genes in 2D and 3D cell cultures.

**Methods:**

*hsa-miR-29c-5p* and *hsa-miR-135b-5p* expression profiles were modulated by transfecting mimics and antimiRs, respectively, in 2D and 3D cell cultures. The expression of the proteins coded by the genes *CDC42, DNMT3A* (target genes of *hsa-miR-29c-5p*) and *APC* (target gene of *hsa-miR-135b-5p*) were measured by Western Blot.

**Results:**

Results showed that mimics and antimiRs transfection significantly altered the expression of both miRNAs, increasing the expression of *hsa-miR-29c-5p* and reducing the expression of *hsa-miR-135b-5p*, especially in the 3D culture of the cell lines. When analyzing the proteins expression, we observed that AGP01 and AGP03 cell lines transfected with mimics had a reduction in the levels of CDC42 and DNMT3A and all three cell lines transfected with antimiRs had an increase in the expression of the protein APC.

**Conclusion:**

We concluded that three-dimensional culture can be a more representative in vitro model that resembles better the in vivo reality. Our results also showed that *hsa-miR-29c-5p* is an important regulator of *CDC42* and *DNMT3A* genes in the intestinal subtype gastric cancer and *hsa-miR-135b-5p* regulates the *APC* gene in both intestinal and diffuse subtypes of GC. Dysregulation in their expression, and consequently in their respectively signaling pathways, shows how these miRNAs can influence the carcinogenesis of different histological subtypes of gastric cancer.

**Electronic supplementary material:**

The online version of this article (10.1186/s12885-018-4980-7) contains supplementary material, which is available to authorized users.

## Background

Gastric Cancer (GC) is the fifth most common type of cancer and the third leading cause of death by cancer worldwide, representing an important issue in public health. Despite the decline in the worldwide GC incidence and mortality rates, South America remains the third leading world area in incidence of this cancer [1, 2].

GC is a multifactorial and complex disease in which genetic, epigenetic and environmental alterations contribute to its development and progression [3]. Significant advances in the understanding of the molecular aspects of such complex disease was made possible due in vitro experimentation in GC cell cultures. The most common cell culture model is growing the cells in monolayer or two-dimensional (2D) culture, however that method does not represent fully the cell-cell signaling or microenvironment features of an in vivo tumor, creating a need of establishing three-dimensional (3D) cultures to minimize the differences between cell culture and the living tissue of a cancer [4, 5].

One of the many molecular mechanisms altered in GC is the differential expression of microRNAs (miRNAs), small noncoding regulatory RNAs (~ 23 nt) that plays important role in the post-transcriptional regulation of gene expression by inhibiting the translation of its target messenger RNA (mRNA) through binding on its 3’ UTR region [6–8].

miRNAs can regulate hundreds of target mRNAs, thus creating a extended and complex regulatory network of several signaling pathways, including those of oncogenes and tumor suppressor genes such as *WNT* and *PI3K* pathways [9–12]. Altered expression of these miRNAs in the tumor consequently modifies the function of its target genes, some of which regulate important cellular mechanisms for the cancer such as growth, proliferation, invasion and metastasis [13–15].

In previous works from our group, we identified through next generation sequencing miRNAs that composed a molecular signature of the gastric antrum and were differentially expressed between GC and the normal tissue, such as *hsa-miR-204*, *hsa-miR-150*, *hsa-miR-29c* and *hsa-miR-135b* [7, 16]. Among the miRNAs differentially expressed, *hsa-miR-29c-5p* and *hsa-miR-135b-5p* were selected to be further investigated, because they were among the most differentially expressed miRNAs and also showed to be progressively dysregulated in the gastric precancerous cascade (Correa’s Cascate) [17].

Both *hsa-miR-29c-5p* and *hsa-miR-135b-5p* play important roles not only in GC but in several types of cancer and are found to be down- and up-regulated, respectively, in gastric adenocarcinoma. *Hsa-miR-29c-5p* regulates genes such as *DNMT3A*, *CDC42*, *RCC2* and *CDK6* and *hsa-miR-135b-5p* regulates genes such as *APC*, *MID1* and *MTCH2* [9, 18–20]. The consequent abnormal expression of the genes regulated by these miRNAs leads to carcinogenesis process, influencing at tumor progression and cellular growth.

Since little is known about the expression of both *hsa-miR-29c-5p* and *hsa-miR-135b-5p* in a three-dimensional condition and the 3D culture seems to resemble better the in vivo conditions of a tumor, there is a need to investigate the role of these miRNAs in such environment to further validate them as biomarkers and our study is the first one to consider the expression of both miRNAs in the 3D environment of gastric cancer cells. In this study, we induced the ectopic expression of *hsa-miR-29c-5p* and inhibited the action of *hsa-miR-135b-5p* in three GC cell lines, both in monolayer and three-dimensional models and, additionally, evaluated the expression of their target genes *DNMT3A*, *CDC42* and *APC*.

## Methods

### Gastric cancer cell lines and miRNA mimics and antimiRs transfection

AGP01, ACP02 and ACP03 cell lines, previously established by our group [21], were cultured in DMEM containing 10% fetal bovine serum at 37 °C in 5% CO_2_. AGP01 is a cell line established from gastric cancer cells present in the ascitic fluid of a patient with intestinal gastric adenocarcinoma, ACP03 is a cell line derived from a primary intestinal subtype gastric adenocarcinomas and ACP02 is a cell line derived from a primary diffuse subtype gastric adenocarcinoma. All culturing conditions were the same for all three cell lines.

All cell lines were also converted from 2D to 3D by magnetic levitation utilizing Bio-Assembler Kit (n3D Biosciences) according to the manufacturer’s instruction. Briefly, 200 μL of magnetic nanoparticles were added in the medium of a 25 cm^2^ flask with at least 80% confluence and then incubated overnight. In the next day, cells were trypsinized, 2 × 10^5^ cells were seeded in each well of a 24-well plate and a magnetic drive was placed on top of the plate to make the cells levitate.

Cells, both in 2D and 3D, were transfected with 100 nM of the following: mirVana™ *hsa-miR-29c-5p* mimics (MC11335), *hsa-miR-135b-5p* miRNA Inhibitor (MH13044) and Negative Control (4464058) (ThermoFisher, Waltham, MA, USA) according to the manufacturer’s instructions. Transfection in 3D cultures were performed after at least 72 h of magnetic levitation. All transfections were performed in triplicates.

### RNA isolation and quantitative reverse-transcription real time PCR

Total RNA was extracted from the cell lines using the High Pure miRNA Isolation Kit (Roche LifeSciences, Penzberg, Germany) according the manufacturer’s instructions. RNA was quantified using a Qubit® 2.0 fluorometer (ThermoFisher Scientific, Waltham, MA, USA) and was then stored at − 80 °C until performing miRNA profiling.

cDNA was synthetized from 10 ng of total RNA using the TaqMan MicroRNA Reverse Transcription kit (ThermoFisher Scientific) following the manufacturer’s instructions. qRT-PCR was performed in a ABI Prism 7500 thermal cycler using TaqMan Universal Master Mix II and TaqMan MicroRNA Assays for *hsa-miR-29c-5p* (001818), *hsa-miR-135b-5p* (002261) and Z30 (001092) according to the manufacturer’s instructions. All Realtime PCR reactions were performed in triplicate and the average of the results was used in subsequent analysis.

To evaluate differences in the expression levels between each group, the comparative Ct method was used [22], and the endogenous control Z30 was utilized to normalize the expression values.

### In silico prediction of *hsa-miR-29c-5p* and *hsa-miR-135b-5p* target genes

To identify possible target genes that are regulated by the two miRNAs, we used two online databases: TargetCompare (lghm.ufpa.br/targetcompare) and miRTarBase (mirtarbase.mbc.nctu.tw). TargetCompare is a database that allows the user to filter miRNAs and its targets that are associated with determined diseases, and we filtered the results that were associated with GC. MiRTarBase is a database that predicts targets that have already been validated by molecular biology techniques.

We only considered relevant to the study the genes predicted by both plataforms that had relation with cancer and had a strong evidence of validation (Luciferase Reporter assays, Western Blot or qPCR) indicated by miRTarBase.

### Western blotting

Western blot analysis was carried as described by Munhoz et al. [23]. Briefly, cells were scrapped and homogenized in cold PBS (supplemented with 1 mM PMSF [phenylmethylsulfonyl fluoride]) and centrifuged at 4 °C for 5 min at 13,000 *g*. The pellets were suspended in RIPA buffer (Sigma Aldrich, St. Louis, MO, USA) according to manufacturer’s instructions. Samples were incubated on ice under agitation for 15 min, and centrifuged for 5 min at 10,000 *g* at 4 °C. Supernatants were used as total protein and stored at − 80 °C. Protein concentration was determined using the Bio-Rad protein reagent.

Proteins were denatured in Laemmli sample buffer including 5% β-mercaptoethanol (Bio-Rad Laboratories Inc., Hercules, CA, USA) for 5 min at 95 °C. Electrophoresis was performed using 7.5% polyacrylamide gel and Bio-Rad Mini-Protean Tetra Apparatus, transferred to a PVDF membrane (Millipore, Billerica, MA, USA). Membrane was blocked for at least 45 min in 10% Tween 20 diluted in Tris-buffered saline (TBS-T) and 5% fat-free milk. After that, the membrane was incubated with rabbit anti-DNMT3A antibody (1:2000; Abcam, Cambridge, UK), rabbit anti-CDC42 antibody (1:10000, Abcam), and rabbit anti-APC antibody (1:1000, Abcam), all of them overnight at 4 °C. Proteins recognized by antibodies were revealed by a chemiluminescent reagent (Pierce™ ECL Western Blotting Substrate, Thermo Scientific) following the manufacturer’s instructions. β-Actin (1:200, Abcam) was used as an internal control of the experiments.

### Statistical analysis

Shapiro-Wilk test was utilized to verify the normality of the distribution of the miRNA expression data. Comparisons between the groups were made utilizing Student’s T test (for parametric analysis).

*P* values < 0.05 were considered statistically significant, all tests and graphics were made using the R statistical package (ver. 3.3.1).

## Results

### *hsa-miR-29c-5p* and *hsa-miR-135b-5p* expressions did not differ between 2D and 3D cultures of the ACP03 cell line

We evaluated the *hsa-miR-29c-5p* and *hsa-miR-135b-5p* expressions in both 2D and 3D models of the cell lines AGP01, ACP02 and ACP03 and observed that all expression profiles followed a Gaussian distribution, so parametric tests were applied to verify differences between groups.

**Fig. 1:**
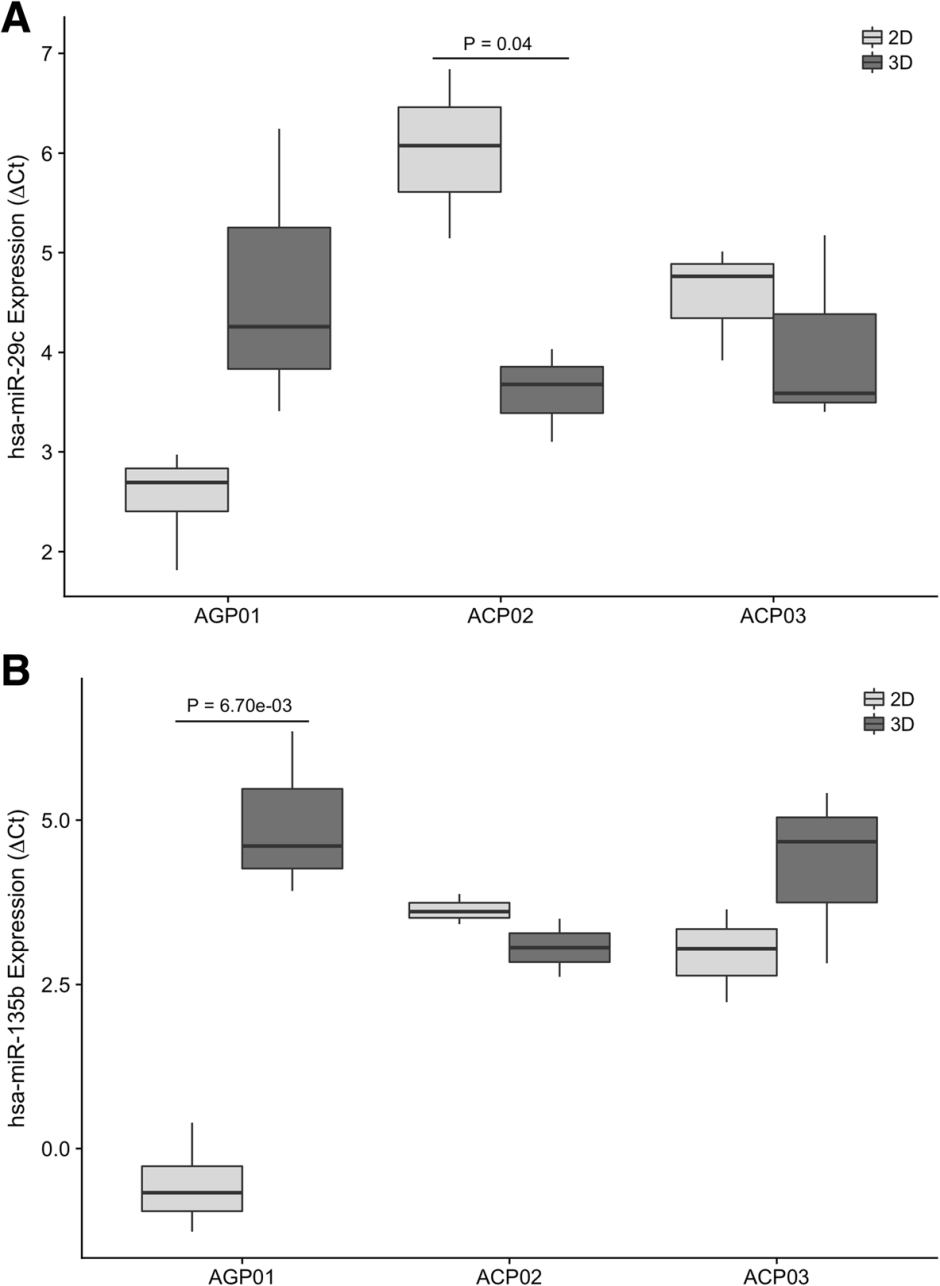
*hsa-miR-29c-5p* (**a**) and *hsa-miR-135b-5p* (**b**) expression profiles in 2D and 3D cultures of AGP01, ACP02 and ACP03 cell lines. (**a**) *hsa-miR-29c-5p* was significantly down-regulated in the 3D culture of ACP02 cell line (*P* = 0.04) and did not differ significantly in the other two cell lines. **b** *hsa-miR-135b-5p* was significantly up-regulated in the 3D culture of AGP01 cell line (*P* = 0.0067) and was not differentially expressed in the other two cell lines. Student’s T test was used to determine statistical significance

When we compared the *hsa-miR-29c-5p* expression between 2D and 3D cultures, we observed that the expression of this miRNA was lower in the 3D model of ACP02 cell line (*P* = 0.04, Fig. 1a), with a fold change of − 4.3. The expression of this miRNA was not statistically different between the two culture models of AGP01 and ACP03 cell lines (*P* = 0.12 and *P* = 0.49, respectively).

As for the *hsa-miR-135b-5p* expression, we observed that the 3D model of the AGP01 had significantly higher levels of this miRNA in comparison to the 2D culture (*P* = 0.0067, Fig. 1b), representing a fold change of 29. ACP02 and ACP03 cell lines showed no significant difference of *hsa-miR-135b-5p* expression between culture models (*P* = 0.14 and *P* = 0.22, respectively). A statistical summary of the results is presented in Additional file 1: Table S1.

### *hsa-miR-29c-5p* mimics and *hsa-miR-135b-5p* antimiRs transfection significantly altered the miRNAs expression profiles in AGP01, ACP02 and ACP03 cell lines

**Fig. 2:**
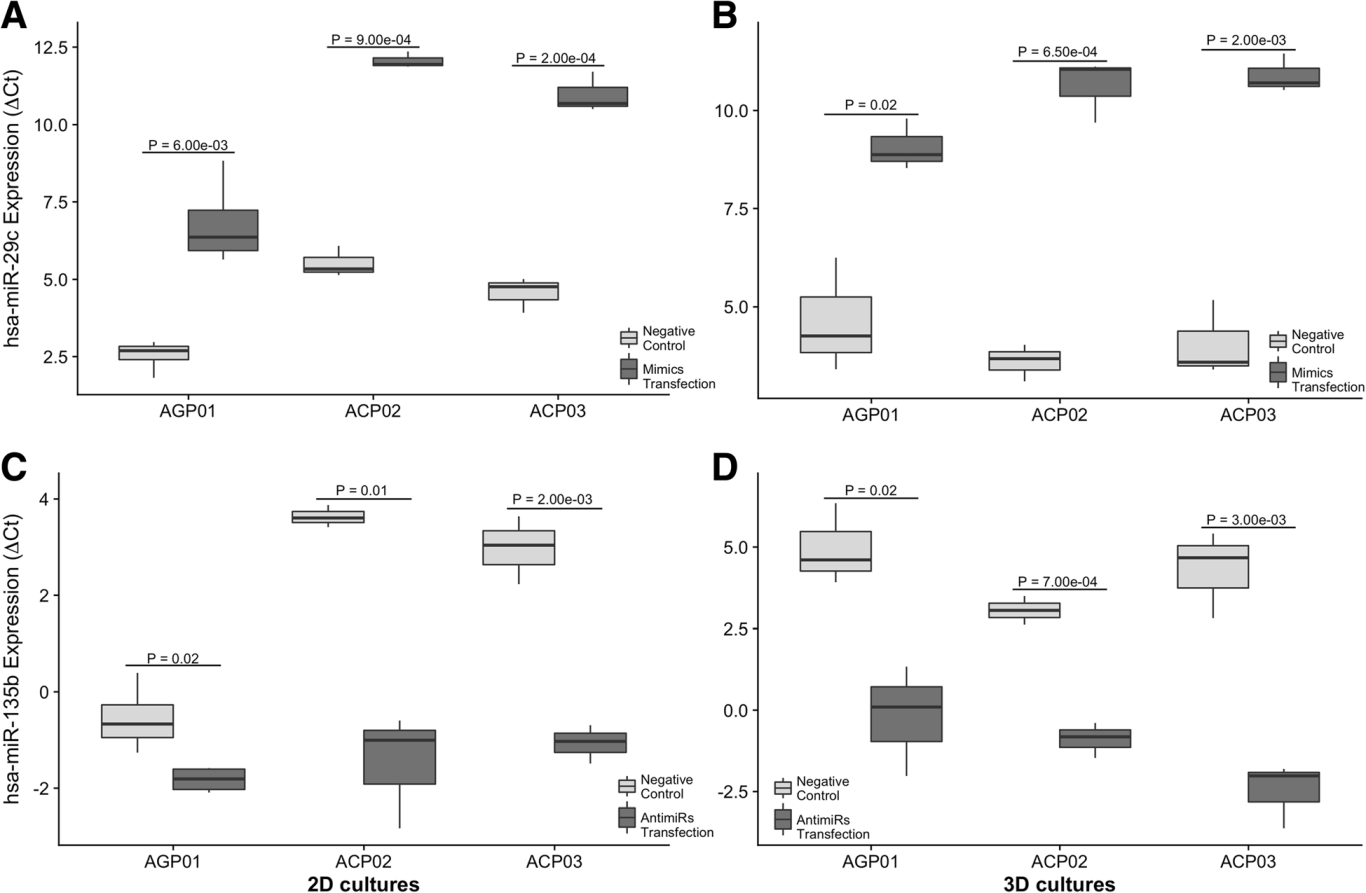
*hsa-miR-29c-5p* (**a**-**b**) and *hsa-miR-135b-5p* (**c**-**d**) expression profiles in control and transfected cells of AGP01, ACP02 and ACP03 cell lines, in both 2D and 3D models. **a**-**b** *hsa-miR-29c-5p* was significantly up-regulated in all three cell lines transfected with mimics in both 2D (**a**) and 3D (**b**) cultures. **c**-**d** *hsa-miR-135b-5p* was significantly down-regulated in all three cell lines transfected with antimiRs in both 2D (**c**) and 3D (**d**) cultures. Student’s T test was used to determine statistical significance

Both miRNAs expressions were measured after 24 h after the transfection of mimics and antimiRs (Fig. 2). We observed that there was a significant increase in *hsa-miR-29c-5p* expression and a significant reduce in *hsa-miR-135b-5p* expression in all three cell lines, both in 2D and in 3D models, when compared to the negative control cells.

In AGP01 cell line, cells that were transfected with *hsa-miR-29c-5p* mimics had a 24-fold increase of this miRNA in the 2D model (*P* = 0.006, Fig. 2a) and a 23-fold increase in the 3D model (*P* = 0.02, Fig. 2b). Cells transfected with *hsa-miR-135b-5p* antimiRs had a − 2.3-fold reduction of the mentioned miRNA in the 2D model (*P* = 0.03, Fig. 2c) and a − 38-fold reduction in the 3D model (*P* = 0.02, Fig. 2d).

In ACP02 cell line, the transfection of mimics increased in 70-fold the *hsa-miR-29c-5p* expression in the 2D model (*P* = 0.0009, Fig. 2a) and 130-fold in the 3D model (*P* = 0.0006, Fig. 2b). Cells transfected with *hsa-miR-135b-5p* antimiRs had its expression reduced in − 30-fold in the 2D model (*P* = 0.015, Fig. 2c) and in − 15-fold in the 3D culture (*P* = 0.0007, Fig. 2d).

Lastly, ACP03 cells that were transfected with mimics had an increase of 87-fold of *hsa-miR-29c-5p* expression in the 2D model (*P* = 0.0002, Fig. 2a) and a 117-fold increased expression in the 3D model (*P* = 0.002, Fig. 2b). *hsa-miR-135b-5p* expression was − 16-fold reduced in 2D cultures transfected with antimiRs (*P* = 0.002, Fig. 2c) and − 100-fold reduced in 3D cultures (*P* = 0.003, Fig. 2d). A statistical summary of the transfection results is presented in Additional file 1: Table S2.

### In silico predictions indicated *DNMT3A* and *CDC42* genes as targets of *hsa-miR-29c-5p* and *APC* as a target of *hsa-miR-135b-5p*

TargetCompare and miRTarBase online tools provided lists with several target genes for both miRNAs, however only the genes that were related to carcinogenesis, were predicted by both platforms and had a strong evidence of validation (Luciferase Reporter Assay, Western Blot and qPCR) according to miRTarBase were considered relevant to our study.

The lists comprised *PDK4*, *PGC-1*α, *SLC39A9*, *XPO5*, *DNMT3A* and *CDC42* as possible target genes of *hsa-miR-29c-5p* and *LPP*, *PPM1A* and *APC* as possible target genes of *hsa-miR-135b-5p*. When we compared these lists of possible target genes with the list of Driver genes of the carcinogenesis from Vogelstein et al. (2013), we observed that *DNMT3A* and *APC* were considered Driver genes and we chose to evaluate their expression after the transfection experiments. *CDC42* was also chosen to be studied because it has a critical role in several metabolic pathways of cancer that leads to proliferation, migration and invasion (Qadir et al., 2015).

### *APC*, *CDC42* and *DNMT3A* had their expressions altered after *hsa-miR-135b-5p* antimiRs and *hsa-miR-29c-5p* mimics transfection

In order to verify if *APC*, *CDC42* and *DNMT3A* had their expressions regulated by *hsa-miR-135b-5p* and *hsa-miR-29c-5p*, we quantified through western blot the expression of the proteins translated by those genes in control cells and in cells transfected with mimics of *hsa-miR-29c-5p* or antimiRs of *hsa-miR-135b-5p*.

**Fig. 3:**
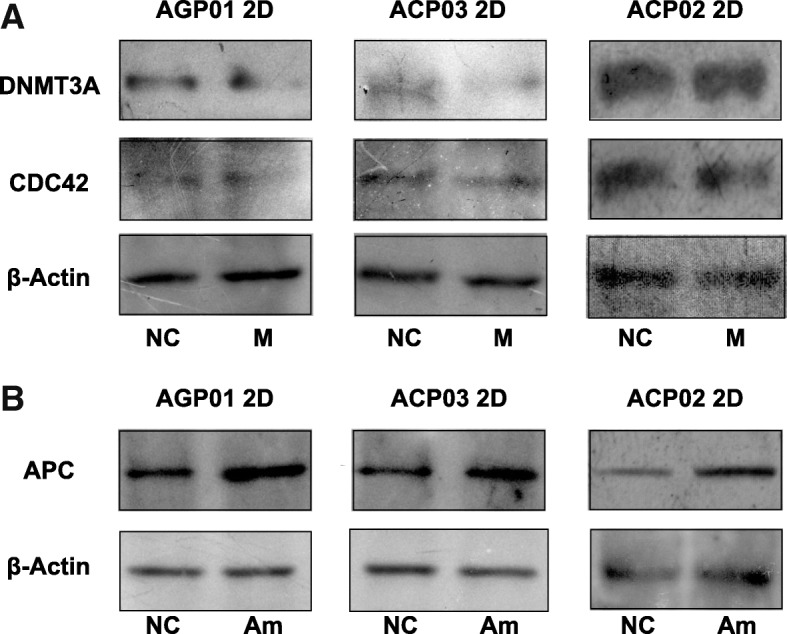
Western Blot of DNMT3A, CDC42 (**a**) and APC (**b**) proteins in control and transfected cells of AGP01, ACP02 and ACP03 cell lines. (**a**) DNMT3A and CDC42 were down-regulated after the transfection of mimics of *hsa-miR-29c-5p* in AGP01 and ACP03 2D cell lines. There was no difference in the expression of both proteins in transfected cells of ACP02 2D cell line. **b** APC was up-regulated in all three 2D cell lines after the transfection of *hsa-miR-135b-5p* antimiRs. β-Actin was used as a loading control in all blot experiments. NC stands for Negative Control; M for transfected with mimics; and Am for transfected with antimiRs

After the transfection with mimics of *hsa-miR-29c-5p*, we observed a reduction in the expression of *CDC42* and *DNMT3A* in AGP01 and ACP03 2D cell lines and no changes between the control and the transfected cells of ACP02 2D cell line (Fig. 3a). In all three 2D cell lines transfected with antimiRs of *hsa-miR-135b-5p* we verified that the expression of *APC* was higher in the treated cells when compared to the control cells (Fig. 3b).

We, however, could not observe expression of any of the three proteins we studied in the 3D cultures of the three cell lines. The proteins did quantify in Bio-Rad protein reagent (Data not shown) but was not possible to obtain data from those cultures in the SDS-PAGE, so we are only showing data obtained from 2D cell cultures (Fig. 3).

## Discussion

Highly sophisticated mechanisms of gene regulation act simultaneously in order to maintain homeostasis and the good functioning of the cell. One of these mechanisms is the gene regulation by miRNAs, a class of small noncoding RNAs that physically repress the translation of their target mRNAs. miRNAs have a tissue-specific expression profile [24] and several studies found that characteristic expression profile to be altered in complex diseases such as cancer [15, 18, 19, 25–29].

Previous studies from our group found that the miRNAs *hsa-miR-29c-5p* and *hsa-miR-135b-5p* were down-regulated and up-regulated, respectively, in gastric cancer when compared to normal gastric mucosa [7, 17] and other studies as well have identified these miRNAs as differentially expressed in other types of cancer [9, 13, 30, 31]. Our aim in this present study was to verify the expression profile of these two miRNAs in 2D and 3D cultures, modulate their expression profile and quantify the expression of its target genes after the transfection with mimics of *hsa-miR-29c-5p* and antimiRs of *hsa-miR-135b-5p*.

According to our previous studies, we expected to found a down expression of *hsa-miR-29c* and an overexpression of *hsa-miR-135b* in cancer samples [7, 17]. In this present study we evaluated the expression profile of miRNAs *hsa-miR-29c-5p* and *hsa-miR-135b-5p* in gastric cancer cell lines, both in 2D and 3D by magnetic levitation models. We found that *hsa-miR-29c-5p* was down-regulated in the 3D culture of ACP02 and *hsa-miR-135b-5p* was up-regulated in the 3D culture of AGP01 (Fig. 1), implying that the three-dimensional conformation of those cell lines influenced those miRNAs expression profile.

3D in vitro models have been widely used in biomedical research since they present characteristics that resembles an in vivo environment, such as the intercellular communication, hypoxia, resistance to drugs, angiogenesis signaling and a tumor microenvironment [32–37].

To further investigate the role of the miRNAs *hsa-miR-29c-5p* and *hsa-miR-135b-5p* in 2D and 3D cultures of gastric cancer, we modulated the expression of both miRNAs in three different GC cell lines and measured the expression of the protein coded by their target genes *DNMT3A*, *CDC42* (for *hsa-miR-29c-5p*) and *AP*C (for *hsa-miR-135b-5p*). As shown in Fig. 2, all cell lines transfected with mimics had an up-regulation of *hsa-miR-29c-5p* of at least 23 fold and cells transfected with antimiRs had a down-regulation of *hsa-miR-135b-5p* of at least − 2.3-fold. After the transfection experiments, we then proceeded to evaluate if the target genes of the two studied miRNAs suffered a major change in their expression as well.

Despite ACP02 and AGP01 3D cultures presented a differential expression of *hsa-miR-29c-5p* and *hsa-miR-135b-5p*, respectively, in relation to their 2D counterpart, we could not observe any protein expression in none of the three cell lines. Total protein lysates of 3D cultures did quantify in Bio-Rad protein reagent but did not separate by molecular weight in the SDS-PAGE, and this result can be explained since the three-dimensional conformation alters the protein composition, specially of extracellular matrix components [38]. This change in protein conformation and composition can be influencing in bonds between proteins making them stronger, leading to a non separation in SDS-PAGE.

In AGP01 and ACP03 2D cultures, cells transfected with mimics had an evident reduction in *DNMT3A* and *CDC42* in relation to the control cells (Fig. 3). In ACP02 2D cell line, we did not observe significant differences in the expression of both genes between transfected and control cells. This can be explained since ACP02 is a cell line originated from a diffuse gastric cancer and this GC subtype is known to have distinct risk factors from the intestinal subtype [39]. So, despite *hsa-miR-29c-5p* being differentially expressed in diffuse cancer, *DNMT3A* and *CDC42* genes are not being regulated effectively by this miRNA and are being activated by another pathway.

The *APC* gene presented an up-regulated expression in the 2D cultures of all three transfected cell lines when compared to the control cells. This result corroborates the findings of Valeri et al. [12] in colorectal cancer and demonstrates that this gene is being actively regulated by *hsa-miR-135b-5p* in both intestinal and diffuse gastric cancer.

Up-regulation of *CDC42* and *DNMT3A* genes and down-regulation of *APC* gene in gastric cancer have several implications in the malignancy of the cell. *APC* is a tumor-suppressor gene and has an important role in the negative regulation of the Wnt signaling pathway, a key pathway that promotes cell proliferation and migration due β-catenin overexpression, which induces the transcription of oncogenes such as c-Myc [40, 41]. APC regulates β-catenin by being a fundamental part of a multi-protein complex that marks it for proteasomal degradation [42]. Thus, down-regulation of *APC* caused by *hsa-miR-135b-5p* enhanced activity leads to a higher transduction signaling within the β-catenin/Wnt pathway, and consequently a higher proliferative capacity of malignant cells.

*APC* loss of function, especially due frameshift and nonsense mutations, or down-regulation is a major cause in the colorectal tumorigenesis and it’s been proposed its loss is sufficient to induce carcinogenesis [42, 43]. Our results indicate that *APC* down-regulation is also an important risk factor to the development of gastric cancer and that this gene may have a crucial function in gastrointestinal malignant neoplasia.

Overexpression of *CDC42* is responsible for the initiation of several biochemical cascades such as cell polarity, cytoskeleton remodeling and migration. Dysregulation in the expression of this gene grants oncogenic activities to the cell and it is associated to cancer development and metastasis [44]. *CDC42* also negatively regulates the expression of p53 and knockdown of *CDC42* via hsa-miR-29 family increased p53-dependent apoptosis in cervical carcinoma cells [45].

Up-regulation of *CDC42* in gastric cancer was observed by Cheng et al. [46] and their study showed that *hsa-miR-133* was down-regulated in GC samples and demonstrated that exogenous expression of this miRNA regulated the *CDC42* levels in GC cell lines.

Since *hsa-miR-29c-5p* and *hsa-miR-133* are down-regulated in gastric cancer [7, 17, 46] and have the same target gene (*CDC42*), it’s possible to observe that independent mechanisms are altered in a similar way to maintain the malignant state of the cell. Our study showed that the exogenous expression of *hsa-miR-29c-5p* is also able to regulate the expression of *CDC42* in intestinal type gastric cancer cell lines, suggesting that this miRNA is another fundamental element in the regulation of this gene.

Up-regulation of *DNMT3A* was already correlated with the down-regulation of *hsa-miR-29c-5p* in cutaneous melanoma and in gastric cancer [9, 31] . Ngyuen et al. [31] observed in cutaneous melanoma that the expression of *DNMT3A* increased as the pathological staging of the disease progressed and the *hsa-miR-29c-5p* expression decreased according to the stage of the disease. These results suggest that dysregulation of both *hsa-miR-29c-5p* and *DNMT3A* occur in the beginning of the carcinogenesis and are important factors to the tumor progression.

Our study investigated the relation between the expression of *hsa-miR-29c-5p* and the expression of *DNMT3A* in three GC cell lines and our results showed that the expression of this miRNA and this gene are inversely correlated. Cui et al. [9] observed that the down-regulation of *hsa-miR-29c-5p* in gastric cancer is due the methylation of the promoter region of the *MIR29C* gene and up-regulation of *DNMT3A* caused higher rates of migration and invasion of malignant cells. The same authors also found that overexpression of *DNMT3A* is responsible for methylating the CpG islands in the *CDH1* promoter region, which lead to E-cadherin down regulation and increased malignancy of gastric cancer cells. *DNMT3A* also down regulates p18^INK4C^ (an important cell cycle regulator and inhibitor of CDK4/6-CyclinD complexes) in a methylation-dependent manner, which leads to increased cell proliferation in gastric cancer [47].

Understanding how the dynamics of gene regulation, via miRNA activity, function within distinct histological subtypes of gastric cancer is essential to elucidate new mechanisms that allow a certain gene or signaling pathway to remain expressed. We observed that *CDC42* and *DNMT3A* remained expressed after the transfection of *hsa-miR-29c-5p* mimics in the diffuse subtype gastric cancer cell line, so investigating what molecular pathways are activated to allow these genes to be constitutively expressed may reveal a unique gene signature of such aggressive cancer and new targets for a more personalized therapeutic approach for patients with that disease.

In silico analysis utilizing miRTarBase (an experimentally validated microRNA-target interactions database - http://mirtarbase.mbc.nctu.edu.tw/php/index.php) showed that both *hsa-miR-135a* and *hsa-miR-135b* shared *APC* as a target gene; we also saw that *hsa-miR-29a*, *hsa-miR-29b* and *hsa-miR-29c* had *CDC42* and *DNMT3A* as target genes. Since in previous works [7] we observed that in samples from our population both *hsa-miR-29c* and *hsa-miR-135b* were the most relevant and differentially expressed miRNAs, we selected those two specific miRNAs from their family members to be studied in the present work.

In vitro and in vivo modulation of miRNAs may represent a promising alternative in the search of new therapeutic targets since they act in critical pathways altered in cancer, specially those that promotes cell proliferation, migration and invasion. Utilizing 3D in vitro models allows a better representation of the spatial conformation observed in vivo, mainly because the composition of the extracellular matrix and a presence of a tumor microenvironment. Hageman et al. [48] showed that not only immortalized cancer cell lines but cells from a primary malignant tumor can undergo 3D spheroids, which allows a high throughput screening method for current or even new therapies to treat cancer in a more efficient and personalized manner.

**Fig. 4:**
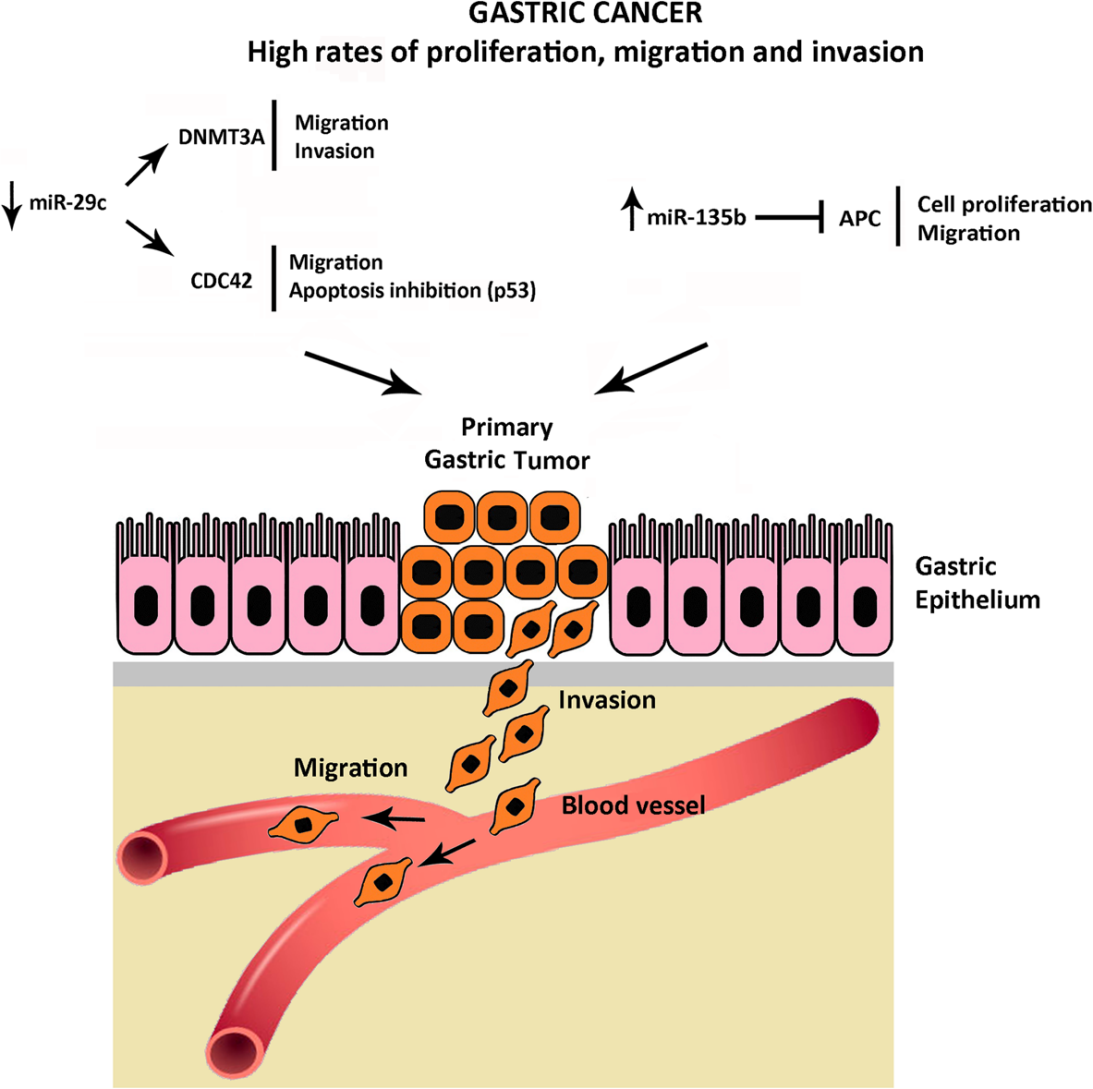
Schematic representation of DNMT3A, CDC42 and APC regulation by *hsa-miR-29c* and *hsa-miR-135b* in Gastric Cancer. Down-regulation of *hsa-miR-29c* and up-regulation of *hsa-miR-135b* leads to inhibition of apoptosis and higher levels of migration, invasion and cell proliferation

Gastric cancer is an aggressive disease with poor rates of survival after diagnosis, and our results suggest molecular pathways that may contribute to such aggressiveness. Therefore, here we suggest an scheme of the possible signaling pathways activated by both *hsa-miR-29c* and *hsa-miR-135b*, through regulation of *DNMT3A*, *CDC42* and *APC* genes. It is possible that the aberrant expression of these miRNAs in GC allows the activation of molecular mechanisms considered to be hallmarks of cancer such as invasion, migration, cell proliferation and inhibition of apoptosis (Fig. 4).

Therapies based in the modulation of miRNAs expression are promising and altering the expression of *hsa-miR-29c-5p* and *hsa-miR-135b-5p*, as well as a higher number of miRNAs can allow an alternative or neoadjuvant therapy to current treatments employed in the management of gastric cancer.

## Conclusions

Cells cultured in a 3D environment showed differences in molecular and morphological aspects from the traditional monolayer method, meaning that this three-dimensional culture can be a more representative in vitro model that resembles better the in vivo reality.

Modulating the expression of the investigated miRNAs caused their target genes to alter their expression in gastric cancer cell lines. *DNMT3A* and *CDC42* genes had its expression down-regulated after the transfection of *hsa-miR-29c-5p* mimics in intestinal subtype cell lines and had no difference in its expression on diffuse subtype cell line, implying that these two genes are fundamental to the more aggressive characteristics of this cancer and are being activated by alternative pathways. *APC* had its expression up-regulated after the transfection of antimiRs of *hsa-miR-135b-5p* in all three GC cell lines, implying that this miRNA is a major regulator of this gene in gastric carcinogenesis.

Interfering in miRNAs expression profiles that act in key signaling pathways responsible for processes such as proliferation, invasion and apoptosis in vitro or in vivo models can reveal new and more personalized therapeutic targets for the management of gastric cancer.

## Additional file

Additional file 1: **Table S1.** Statistical summary of the miRNAs *hsa-miR-29c-5p* and *hsa-miR-135b-5p* expression profiles between 2D and 3D cell cultures. **Table S2.** Statistical summary of the transfection experiments of *hsa-miR-29c-5p* mimics and *hsa-miR-135b-5p* antimiRs in AGP01, ACP02 and ACP03 cell lines, both in 2D and in 3D models in relation to its negative control counterpart. (DOCX 20 kb)

## Abbreviations

2D: Two-dimensional
3D: Three-dimensional
GC: Gastric cancer
miRNAs: microRNAs
