# Supplementary material for: APC gene is modulated by hsa-miR-135b-5p in both diffuse and intestinal gastric cancer subtypes
Source: BMC Cancer. 2018 Oct 30;18:1055. doi: 10.1186/s12885-018-4980-7 (PMC6208123; doi:10.1186/s12885-018-4980-7)
Supplement: Supplementary file 1 — Table S1. Statistical summary of the miRNAs hsa-miR-29c-5p and hsa-miR-135b-5p expression profiles between 2D and 3D cell cultures. Table S2. Statistical summary of the transfection experiments of hsa-miR-29c-5p mimics and hsa-miR-135b-5p antimiRs in AGP01, ACP02 and ACP03 cell lines, both in 2D and in 3D models in relation to its negative control counterpart. (DOCX 20 kb) [file 12885_2018_4980_MOESM1_ESM.docx]

**Supplementary Table 1:** Statistical summary of the miRNAs *hsa-miR-29c-5p* and *hsa-miR-135b-5p* expression profiles between 2D and 3D cell cultures.

| miRNA | Fold change | Cell line | P-value* |
| --- | --- | --- | --- |
|  | 2.21 | AGP01 | 0.12 |
| *hsa-miR-29c-5p* | -4.3 | ACP02 | 0.04 |
|  | -0.98 | ACP03 | 0.49 |
|  | 29 | AGP01 | 0.0067 |
| *hsa-miR-135b-5p* | -1.55 | ACP02 | 0.14 |
|  | 2,28 | ACP03 | 0.22 |

(*) P-*value* obtained by performing Student's T test.

**Supplementary Table 2:** Statistical summary of the transfection experiments of *hsa-miR-29c-5p* mimics and *hsa-miR-135b-5p* antimiRs in AGP01, ACP02 and ACP03 cell lines, both in 2D and in 3D models in relation to its negative control counterpart.

| miRNA transfected | Cell line | Culture model | | Fold change | P-value* |
| --- | --- | --- | --- | --- | --- |
| *hsa-miR-29c-5p mimics* | AGP01 | | 2D | 24 | 0.006 |
|  |  |  | 3D | 23 | 0.02 |
|  | ACP02 | | 2D | 70 | 0.0009 |
|  |  |  | 3D | 130 | 0.0006 |
|  | ACP03 | | 2D | 87 | 0.0002 |
|  |  |  | 3D | 117 | 0.002 |
| *hsa-miR-135b-5p antimiRs* | AGP01 | | 2D | -2.3 | 0.03 |
|  |  |  | 3D | -38 | 0.02 |
|  | ACP02 | | 2D | -30 | 0.015 |
|  |  |  | 3D | -15 | 0.0007 |
|  | ACP03 | | 2D | -16 | 0.002 |
|  |  |  | 3D | -100 | 0.003 |

(*) P-*value* obtained by performing Student's T test.
